# Supplementary figures and images for: Transmission Patterns of Co-Circulation of Omicron Sub-Lineages in Hong Kong SAR, China, a City with Rigorous Social Distancing Measures, in 2022
Source: Viruses. 2024 Jun 18;16(6):981. doi: 10.3390/v16060981 (PMC11209396; doi:10.3390/v16060981)

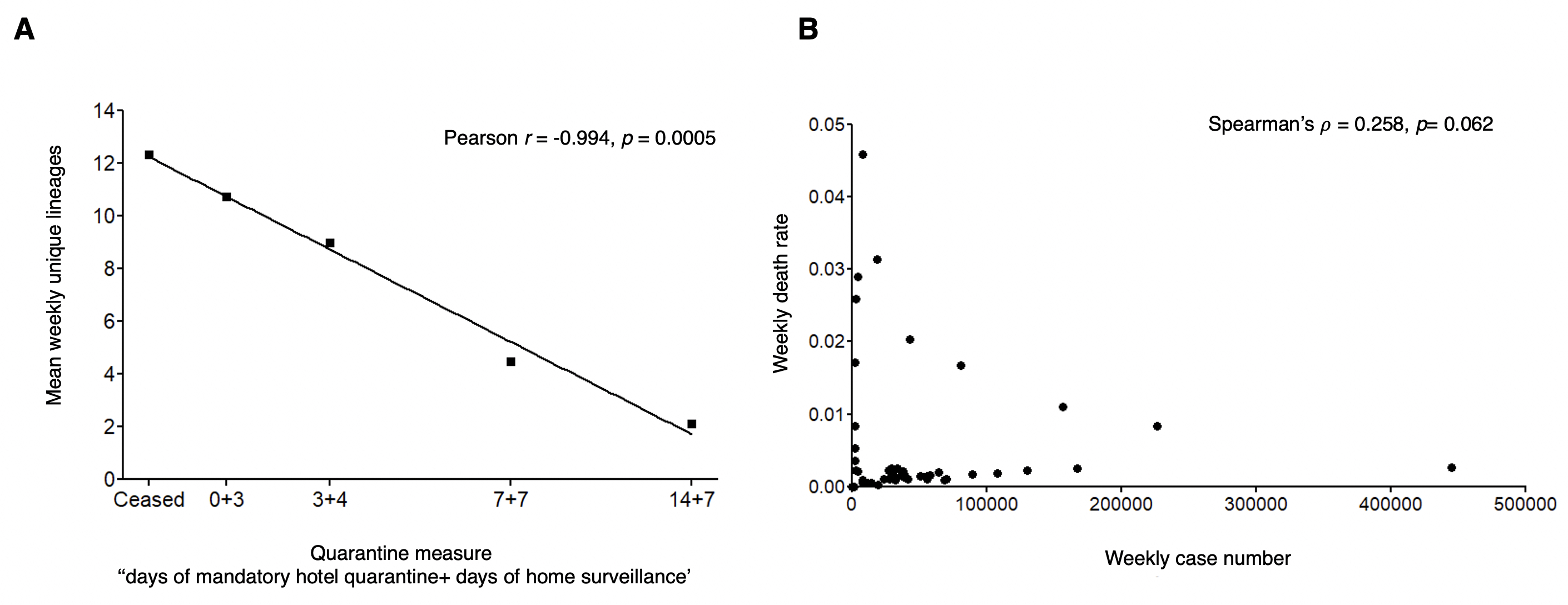

Supplement: Supplementary file 1 [file viruses-16-00981-s001.zip › Figure S2.jpeg]

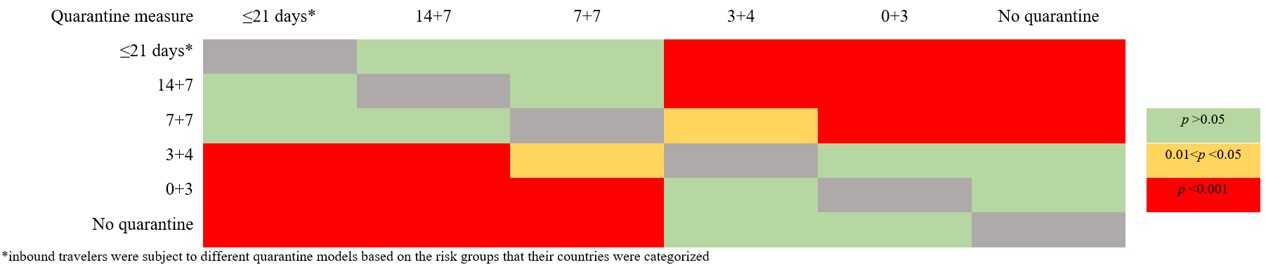

Supplement: Supplementary file 1 [file viruses-16-00981-s001.zip › Figure S3.jpg]

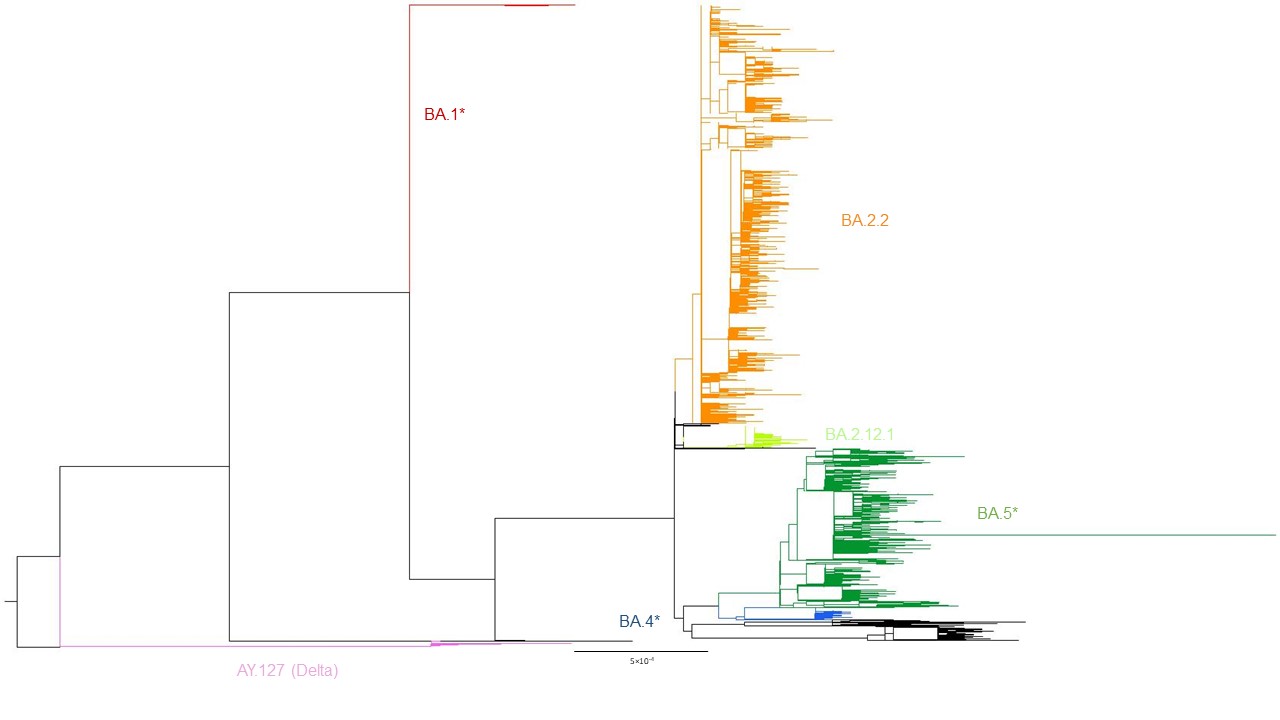

Supplement: Supplementary file 1 [file viruses-16-00981-s001.zip › FigureS1.jpg]
